# On the Choice of Longitudinal Models for the Analysis of Antitumor Efficacy in Mouse Clinical Trials of Patient-derived Xenograft Models

**DOI:** 10.1158/2767-9764.CRC-22-0238

**Published:** 2023-01-26

**Authors:** Hélène Savel, Sandrine Barbier, Cécile Proust-Lima, Virginie Rondeau, Rodolphe Thiébaut, Florence Meyer-Losic, Laura Richert

**Affiliations:** 1 Department of Public Health, Inserm Bordeaux Population Health Research Centre, U1219, University of Bordeaux, Bordeaux, France.; 2 Ipsen Innovation, Les Ulis, France.; 3 Inria, SISTM, Talence, France.; 4 University of Bordeaux, INSERM, CHU de Bordeaux, Institut Bergonié, CIC-EC 1401, Bordeaux, France.

## Abstract

In translational oncology research, the patient-derived xenograft (PDX) model and its use in mouse clinical trials (MCT) are increasingly described. This involves transplanting a human tumor into a mouse and studying its evolution during follow-up or until death. A MCT contains several PDXs in which several mice are randomized to different treatment arms. Our aim was to compare longitudinal modeling of tumor growth using mixed and joint models.

Mixed and joint models were compared in a real MCT (*N* = 225 mice) to estimate the effect of a chemotherapy and a simulation study. Mixed models assume that death is predictable by observed tumor volumes (data missing at random, MAR) while the joint models assume that death depends on nonobserved tumor volumes (data missing not at random, MNAR).

In the real dataset, of 103 deaths, 97 mice were sacrificed when reaching a predetermined tumor size (MAR data). Joint and mixed model estimates of tumor growth slopes differed significantly [0.24 (0.13;0.36)log(mm^3^)/week for mixed model vs. −0.02 [−0.16;0.11] for joint model]. By disrupting the MAR process of mice deaths (inducing MNAR process), the estimate of the joint model was 0.24 [0.04;0.45], close to mixed model estimation for the original dataset. The simulation results confirmed the bias in the slope estimate from the joint model.

Using a MCT example, we show that joint model can provide biased estimates under MAR mechanisms of dropout. We thus recommend to carefully choose the statistical model according to nature of mice deaths.

**Significance:**

This work brings new arguments to a controversy on the correct choice of statistical modeling methods for the analysis of MCTs. We conclude that mixed models are more robust than joint models.

## Introduction

In the preclinical phase of oncology drug development, various *in vitro* and *in vivo* models can be used to evaluate the antitumor efficacy of a compound. Compared with tumor models based on established cell lines, patient-derived xenografts (PDX) implanted into immunocompromised mice are derived from patient's tumors and better recapitulate the heterogeneity and genetic diversity observed in patients. These models have the ability to bridge the gap between the homogeneity of preclinical data generated on cell line–derived mouse models and the heterogeneity of human tumors observed in clinical trials (1).

**FIGURE 1:**
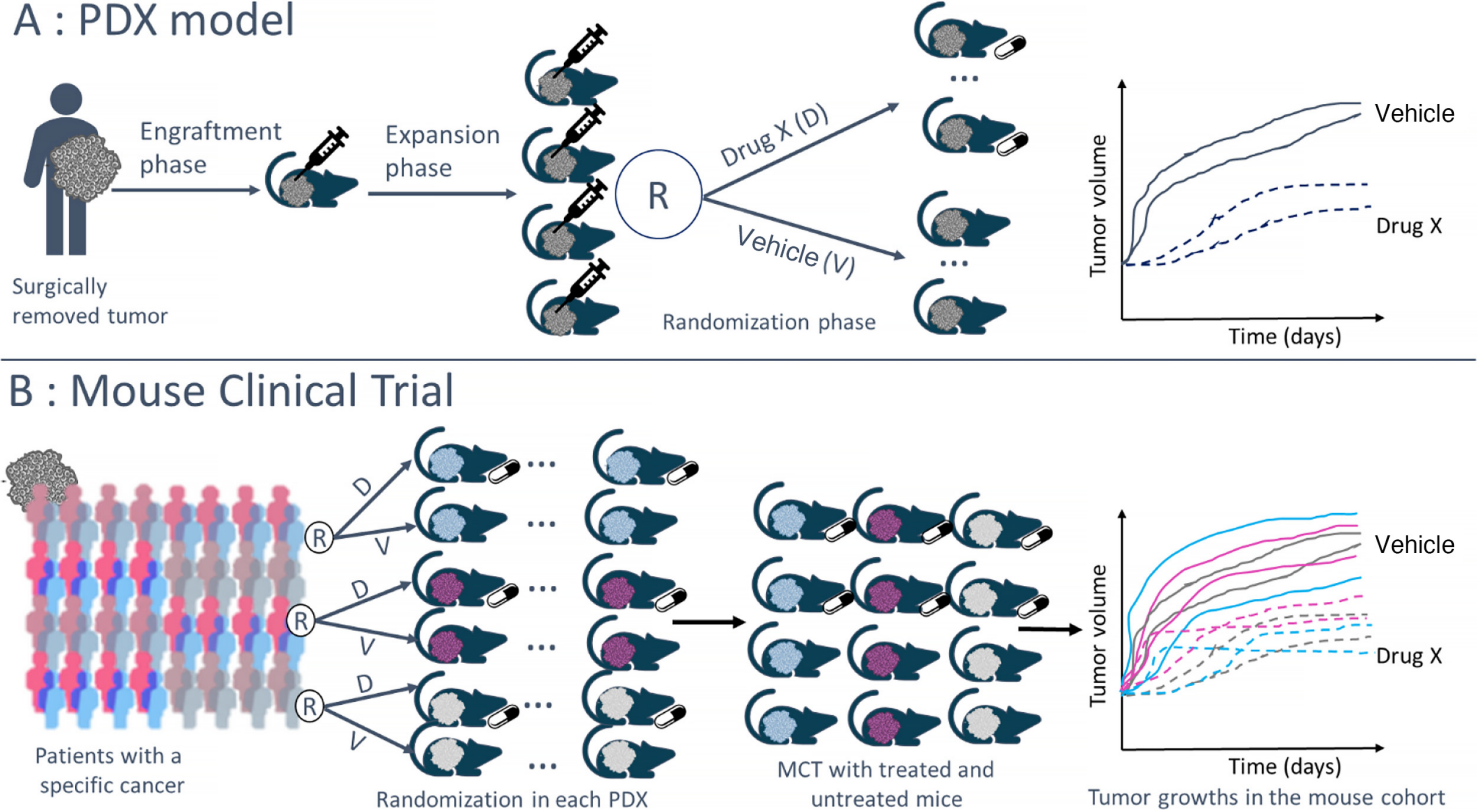
Description of PDX model and MCT design. **A,** PDX model. **B,** MCT design.

Moving beyond the classical efficacy study with PDX models to estimate antitumor efficacy for a single tumor model, there is an innovative and recent preclinical experiment design, the mouse clinical trial (MCT) or PDX Clinical Trial (2, 3), which allows the estimation of treatment response in a pooled analysis of several PDX models (i.e., with tumors from different human patients). This type of design makes it possible to mimic more closely the populational inference approach taken in clinical studies and improve translational approaches from preclinical to early clinical results (3). In MCTs, each PDX can include several mice that are randomized within the PDX model, that is, for each PDX, between 1 and *n* mice from this model are randomized between a vehicle control group and the different treatment arms studied. At the end of the trial, results of several mice from different PDX models are observed in each treatment arm as described in Fig. 1.

In preclinical oncology models, antitumor efficacy is usually measured by the dynamic of tumor growth in response to a defined treatment. For each PDX model, after an expansion phase, this dynamic of the subcutaneous tumor size of each mouse is measured with calipers over a given follow-up period or until the death of the mouse if this occurs before the end of the planned follow-up.

How to best analyze the data collected in such MCT experiments has not clearly been resolved. In the literature, several criteria have been suggested to summarize this dynamic (Supplementary Data S1): both continuous (e.g., tumor growth inhibition, AUC ratio) or categorical [e.g., modified RECIST (mRECIST)] criteria have been suggested (2). However, categorization from quantitative information induces an inherent loss of information. For example, mRECIST is a summary measure of the complete kinetics of tumor growth. In the case of MCTs, it should be noted that even the suggested continuous criteria are in fact summarized measurements of tumor growth kinetics. Some authors recommend using several criteria and then critically evaluate the results obtained (4, 5).

**FIGURE 2:**
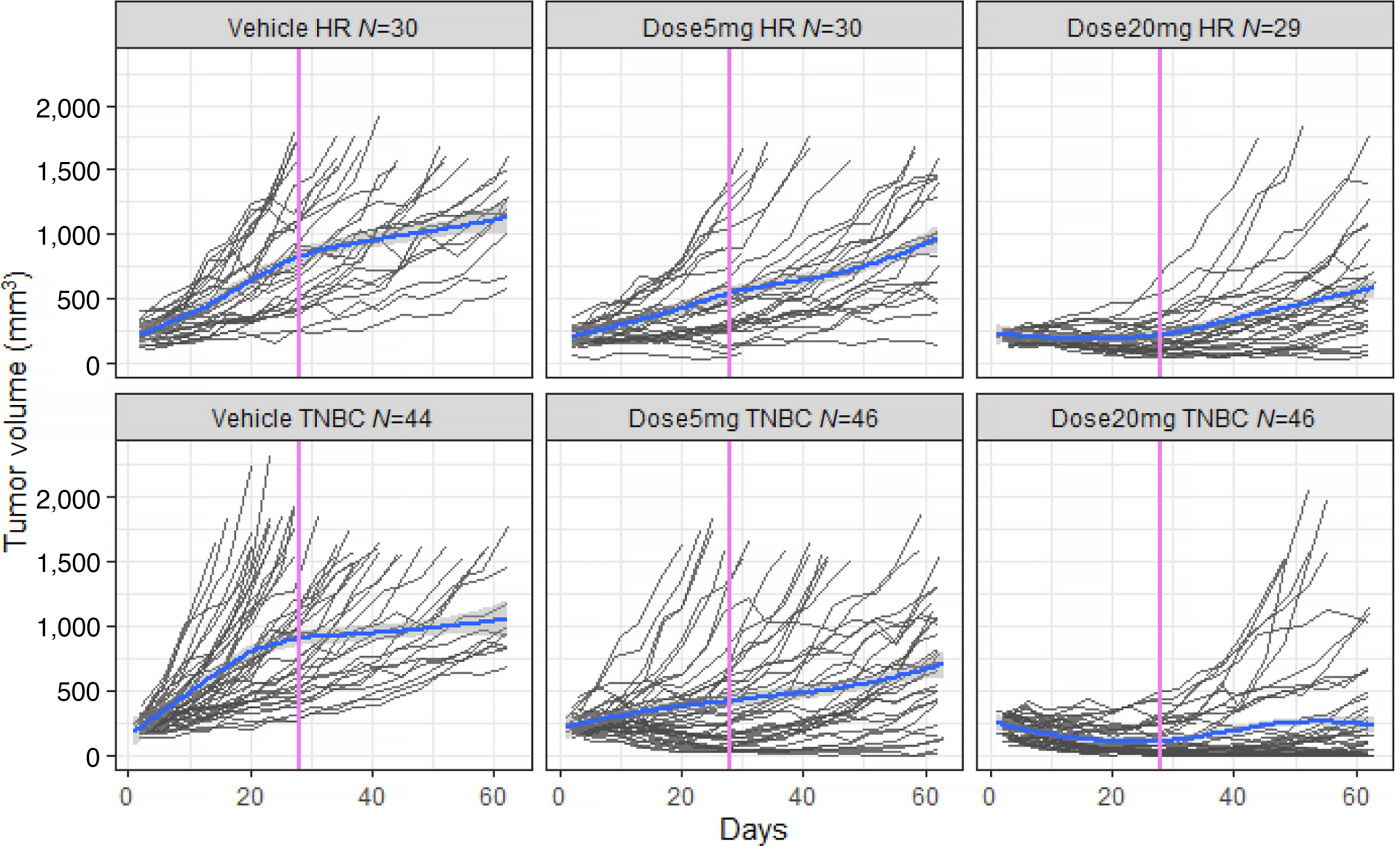
Description of tumor growth evolution by subtypes and doses. The average trend (in blue) and its confidence interval (in gray) are estimated by a Loess-type regression method that ignores missing data mechanisms and within-mouse correlations.

Nevertheless, the most genuine approach is the analysis of quantitative tumor growth evaluated at different times during the follow-up. A simple and often used, but naïve method for the analysis of PDX data could be the comparison of tumor volume between the treatment and control groups at each evaluation time. However, this method is flawed as it does not take into account the shape of the tumor growth and the deaths of mice during follow-up. Because of deaths occurring before the end of follow-up, the evaluation of the treatment effect at late follow-up times can be biased, a well-known concept in epidemiology that is called survivor bias (subtype of selection bias). Indeed, in the presence of many deaths, the comparison between the groups based on observed data at a given timepoint is only made on mice that are still alive, that is, mice that have survived up to this timepoint (and are thus observable). These mice correspond in general to those animals with a less rapid tumor growth (Fig. 2). The evaluation of the effect of the treatment observed on these mice can therefore not be generalized to all the mice included in the experiment, and this simple analysis approach cannot be recommended.

In addition, some authors (2, 6) have shown that comprehensive analysis of tumor growth kinetics using longitudinal statistical models is the most powerful approach to analyze PDX data. To tackle the problem of the deaths of mice during the follow-up, two types of statistical models can be considered: mixed-effects regression models and joint models of tumor growth and survival (7, 8). Both have previously been recommended in the literature for MCT data analysis (2, 9). Yet, the two methods assume different mechanisms of missing tumor volumes induced by early deaths, and do not necessarily give concordant results (10). Mixed models assume that death is predictable by the observed tumor volumes while the joint models assume that death depends on the nonobserved tumor volumes.

Our objective was thus to apply and contrast these two kinds of longitudinal modeling approaches of tumor growth to estimate the antitumor efficacy in the presence of early dropout due to death, with the perspective of providing recommendations on which statistical approach to favor in MCT designs. We focused on MCT designs including several biomarker subgroups because identification of subgroups with promising treatment effects is an important objective when using MCTs as a translational model towards the clinical development.

## Materials and Methods

Two statistical models of tumor growth based on linear mixed-effects regression model on the one hand and a joint shared random-effects model of tumor growth and survival on the other hand were used to analyze a MCT dataset.

### MCT Study

The MCT study was performed at OncoDesign. All experimental procedures were approved by the Ethics Committee of OncoDesign (C2EA; registration number 91) and were performed in full compliance with the ARRIVE guidelines, EU Directive 2010/63/ EU for animal experiments, and the 2013 French Regulatory Decree. All efforts were made to minimize animal suffering and to reduce the number of animals used (11).

The MCT dataset was composed of 25 PDX breast cancer models provided by the IMODI French consortium (12). A total of 225 mice were randomized to three arms with a randomization within each PDX model. The original objective of this MCT was to estimate and compare the effect of two dose levels (5 and 20 mg/kg) of liposomal irinotecan versus vehicle in two subgroups defined according to three tumor markers HER2 (human epidermal growth factor receptor 2), ER (estrogen receptor), PR (progesterone receptor):

- HR for hormonal receptor subgroup (ER and/or PR^+^ and HER2^−^) included 10 PDX with 89 mice
- TNBC for triple-negative breast cancer subgroup (ER^−^, PR^−^, and HER2^−^) included 15 PDX with 136 mice

The 63-day follow-up included an on-treatment period (with three administrations 7 days apart) and then no treatment from day 14 onward. For each mouse, tumor size was measured using calipers approximately every 3 days during follow-up.

Liposomal irinotecan is a chemotherapy drug (cytotoxic agent) whose active ingredient is irinotecan (a topoisomerase I inhibitor) encapsulated in a liposome. Irinotecan and its active metabolite SN-38 inhibits topoisomerase I, a crucial enzyme in DNA replication. As a result, religation of DNA single-strand breaks is prevented, leading to exposure time-dependent, double-stranded DNA damage and cell death (13, 14). In combination with 5-fluorouracil/leucovorin, liposomal irinotecan has been approved for the treatment of patients with metastatic Pancreatic Ductal Adenocarcinoma (mPDAC) following progression with gemcitabine-based therapy [Ipsen Pharmaceuticals Inc. *ONIVYDE (irinotecan liposome injection),* for intravenous use]*. Prescribing information.* June 2017 [cited 2020 8 July]; Available from https://www.ipsen.com/websites/Ipsen_Online/wpcontent/uploads/sites/9/2019/01/21083350/ONIVYDE_USPI.pdf.

### Statistical Modeling Strategy

A log transformation of tumor volume was used to assume an exponential model which is often used to model tumor growth and allows to obtain a Gaussian distribution after transformation.

### Missing Data Mechanisms

In MCT, the tumor growth process is stopped by death (regardless of the cause of death) which entails a dropout and missing tumor volumes. Before applying any statistical method, it is crucial to understand the nature of the missing data as statistical models are not all robust to the same type of missing data mechanism. Two different mechanisms are to be distinguished (15):

- Missing at random (MAR): occurrence of missing data is only related to observed data (in the MCT context, some sacrifices of mice are linked to an ethical threshold of tumor volume not to be exceeded (e.g., 1,500 mm^3^) and tumor volumes are measured by researchers). It is a noninformative study dropout.
- Missing not at random (MNAR): occurrence of missing data is related to unobserved data (in MCT context, death due to natural causes). It is an informative study dropout.

In our use case, after identifying the missing data process related to deaths, an additional analysis was performed by perturbing the initial dataset to modify the missing data process. Taking into account the observed process, the data were disrupted to have a scenario with only MNAR data by making the last TV measurements unobserved before death. This was done by considering only tumor volume measurements below a threshold that is itself below the ethical threshold. The number of deaths and date of deaths were unchanged in this dataset. The objective of these analyses was to assess the robustness of the estimates provided by the mixed and joint models in the MCT framework.

### Linear Mixed-effects Regression Model

A linear mixed-effects regression model, such as recommended by authors (2) for analyses of MCT, is robust to MAR process due to death. With this type of model, the mean evolution of tumor growth is estimated using a linear regression while taking into account the correlation between the repeated tumor volumes of each mouse using mouse-specific correlated random effects on time functions. In the presence of subgroups and different treatments, it is possible to estimate the mean evolution for each subgroup and each treatment. In the case of the MCT of breast cancer treated with liposomal irinotecan, the model included a piecewise linear tumor growth trajectory (one slope on-treatment and a second slope after the end of treatment period) at the population and mouse levels as follows:



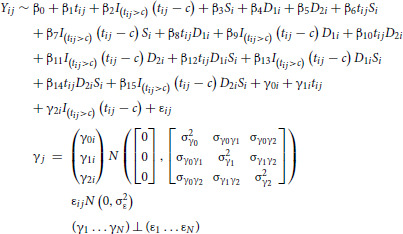



with *i* = 1,…, 225 mice, *j* = 1, …, *n*_*i*_ measured at times *t*_*ij*_, *t*: week, *c*: time of change in slope, S: subgroup (HR/TNBC), D1: dose 5 mg/kg, D2: dose 20 mg/kg.

Several change points were tested (3, 4, and 5 weeks post treatment initiation; Supplementary Data S2). The parameters of the model were estimated by maximum likelihood. In addition, to consider the potential correlation between mice from the same PDX, a nested random effect (mouse effect nested within the PDX effect) on the intercept and both slopes were tested. Indeed, the tumor of mice associated with the same PDX are all derived from the same patient's tumor, thus having correlated tumor characteristics and growth dynamics. In contrast, 2 mice from two different PDXs are less correlated. If biologically relevant and statistically significant, the addition of a nested random effect (two-level random effect) could provide a better estimate of the variability of the data.

These models were implemented with the package lme4 (16) in the R software version 4.1.0 (R Project for Statistical Computing, RRID:SCR_001905).

### Joint Shared Random-Effects Model

Joint shared random-effects models (7) have been proposed to handle longitudinal data in the presence of a MNAR process due to death. The advantage of this type of model is that it takes into account the correlation between the markers under study, in this case tumor volume, and the occurrence of an event during follow-up, in this case the death of the mice. For this purpose, the above mixed model was combined with a proportional hazards model in which the current value of tumor size predicted by the mixed model is included as an explanatory variable to account for the association between the evolution of the marker and the risk of death as follows:

Survival model for the time of event T^*^_i_, *i* = 1, …., 225 was defined as follows:



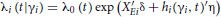



With *X*_*Ei*_, vector of covariates, *h*_*i*_(γ_*i*_, *t*), function of the random effects and η, association parameter with the biomarker.

In our case with current true level 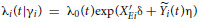


with 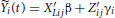


The estimation of the model parameters was done by maximum likelihood using package JM (7) in the R software version 4.1.0 (R Project for Statistical Computing, RRID:SCR_001905). Random effects were considered only at the mouse level because the package did not handle nested random effects.

For these two types of statistical model, the convergence and the accuracy (residuals analysis for linear mixed model part) were checked.

### Simulations

A simulation study was carried out to evaluate the impact of the proportion of MAR and MNAR deaths on the estimation of the parameters of mixed and joint models. In these simulations, we assumed a single group with a 28-day follow-up with several sample size of mice (300, 200, and 100). In addition, a proportion of all-cause deaths of 50% was assumed with the following MNAR and MAR distribution scenarios: 50% MNAR and 0% MAR, 40% MNAR and 10% MAR, 30% MNAR and 20% MAR, 20% MNAR and 30% MAR, 10% MNAR and 40% MAR, 0% MNAR and 40% MAR. For each scenario, 1,000 datasets were created. Tumor growth kinetics were simulated using a single slope mixed linear regression model with random intercept and slope. MNAR deaths were simulated using R programs provided by Thomadakis and colleagues (10) and MAR deaths using log tumor volume thresholds depending on the desired proportion of deaths, which mimic the ethical threshold of real MCTs. These datasets were analyzed with mixed linear regression model with random intercept and slope and with a joint model as described in the previous section.

All simulations and analyses were carried out with the R software version 4.1.0 (R Project for Statistical Computing, RRID:SCR_001905).

### Data Availability Statement

Qualified researchers may request access to study data that underlie the results reported in this publication. Additional relevant study documents, including the clinical study report, study protocol with any amendments, annotated case report form, statistical analysis plan and dataset specifications may also be made available. In case that patient-level data are requested, these will be anonymized, and study documents will be redacted to protect the privacy of study participants.

## Results

### Description of MCT

The real MCT dataset was composed of 25 PDX breast cancer models with a total of 225 mice (between 1 to 18 measures per mice, 75% with at least 11 measures during follow-up) distributed by subtypes and doses as described in Fig. 2. It should be noted that the graphical description of the data is essential but that over time, due to the deaths of the mice, the average representation of the tumor evolution is not representative of the average tumor evolution of the initial study population because it is calculated only on survivors (i.e., mice with low tumor growth evolution). This is an illustration of survivor bias.

**FIGURE 3:**
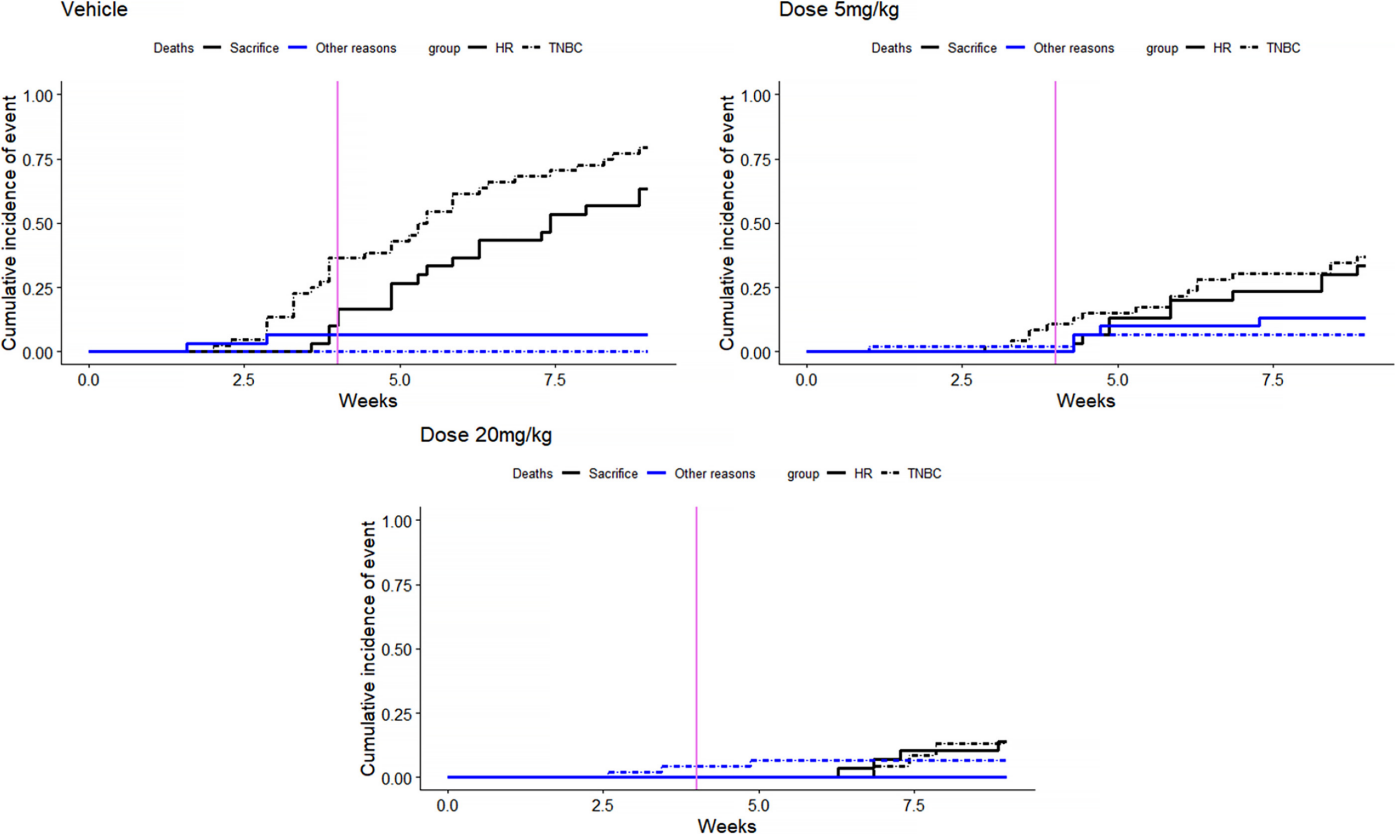
Description of causes of deaths by subtypes and doses. Description by cumulative incidence function.

During the follow-up period, 103 deaths were observed and the reasons and description of deaths (Fig. 3) by subtypes and treatment doses are as follows:

- sacrifice (tumor volume> ethical criteria): *n* = 91
- other reasons [natural (*n* = 2), sacrifice (moribund; *n* = 5), body weight change > ethical criteria (*n* = 1), necrosed tumor (*n* = 2), death after manipulation (*n* = 2)]: *n* = 12

Most deaths (88%) were related to sacrifice after reaching a too large tumor size, which is characteristic of MAR data. Indeed, in this case, the deaths of mice depend on the tumor volume measurement observed at the previous time.

### Statistical Modeling Strategy

Concerning the modeling strategy of the mixed model, the final model was the model with a change of slope at week 4 and random effects on the intercept and the two slopes (Supplementary Data S2). The nested random effect was also tested and was significant (Supplementary Data S3). The analysis of the residuals showed a good fit for both mixed and joint models (Supplementary Data S4).

**FIGURE 4:**
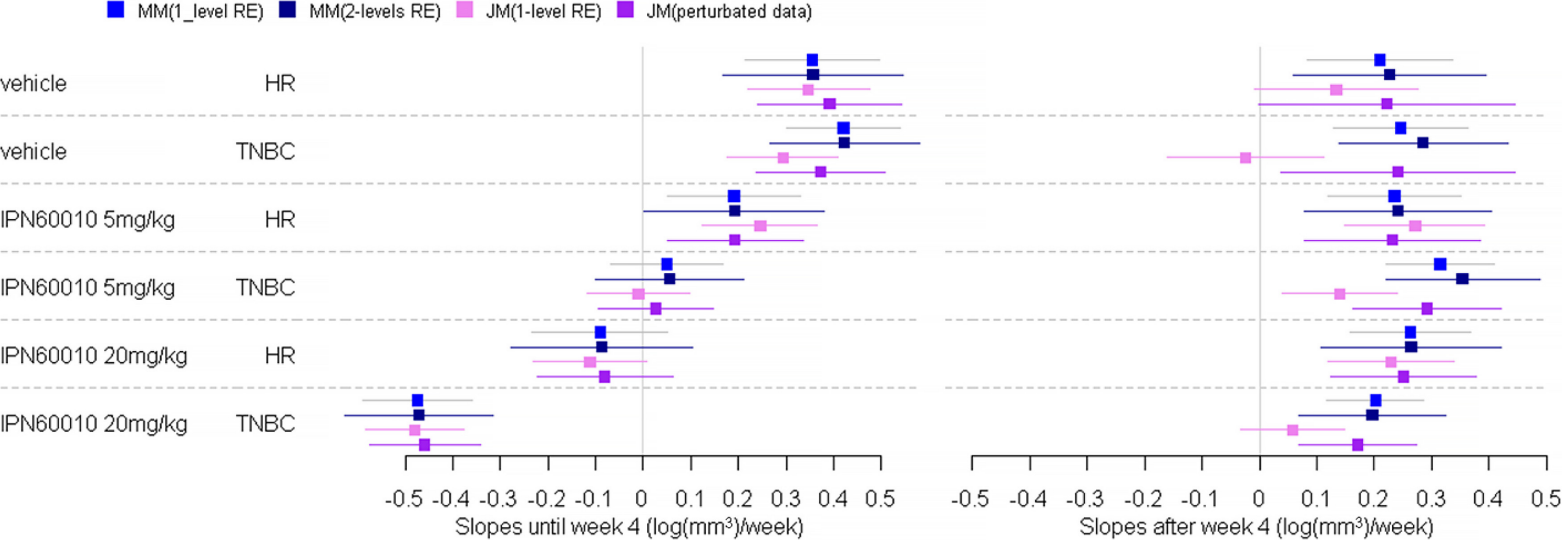
Estimation of treatment effect on tumor growth (log(mm^3^)/week) by doses and subgroups by linear mixed models MM (one-level and two-level random effects RE) and joint modeling JM of tumor growth and death in mice (original dataset and perturbated data). Estimation of slopes and 95% confidence interval per doses and subgroups.

The estimates of the treatment effect by subtypes and doses with three different models (piecewise linear mixed model with one-level random effects, piecewise linear mixed model with two-level random effects and joint model with one-level random effects) are summarized in Fig. 4.

Although it is preferable to take into account all levels of correlation (PDX and mouse), mixed models taking into account only the mouse level correlation (one-level, AIC: 1606.1) gave very similar results to mixed models including also the PDX level correlation (two-level, AIC: 1458.7).

The estimates obtained by joint model under the assumption of MNAR dropout due to death differed substantially from those obtained by the mixed models under the assumption of MAR dropout, especially in the second period when the number of deaths was increasing (Fig. 3). As most of the dropouts were due to sacrifices, that is a MAR process, this illustrates the bias that can be induced by an incorrect assumption regarding the nature of the dropout mechanism.

Furthermore, to confirm that this difference in estimation between the mixed model and the joint model is due to the nature of the deaths, a complementary analysis was performed by perturbating the MAR data process. In the case of MCT and a MAR missing data process on tumor growth, this process can become MNAR by artificially rendering unobserved the tumor values that gave rise to the sacrifice (considering only the tumor volume measurements at an earlier stage than the ethical threshold, 900 mm^3^ for this use case). In this case, mice have between 1 to 17 measures during follow-up and 75% of mice have at least 7 measures. However, the original date of death of each mouse has been retained. The results of these complementary analyses are presented in Fig. 4. As can be seen from the graphs when the missing data process is no longer MAR then the estimates of the joint model and the mixed model on the whole dataset are close. These results support the fact that the biased estimates provided by the joint model on the raw data are likely due to the MAR process of the missing data.

### Results of the Simulation Study

The simulation study showed that, whatever the sample size and the proportion of MAR and MNAR deaths, the mixed model converged more often than the joint model (Table 1). Moreover, the joint model slope estimates were more biased when there was a competitive risk of MNAR and MAR deaths.

**TABLE 1:** Estimation of treatment effect (slope) by mixed and joint models of the simulation study

	Slope (true value : 0.39(log(mm^3^)/week))							
	Mixed model				Joint model			
	Mean	Bias (%)	Coverage (%)	Convergence (%)	Mean	Bias(%)	Coverage(%)	Convergence (%)
***N* = 300**								
50% MNAR 0% MAR	0.39	−0.06	94.4	99.5	0.39	−0.4	94.3	92.1
40% MNAR 10% MAR	0.39	−0.05	94.6	99.4	0.38	−1.1	93.8	74.4
30% MNAR 20% MAR	0.39	−0.04	95.6	99.8	0.34	−13.2	17.2	54.0
20% MNAR 30% MAR	0.39	−0.01	94.5	99.7	0.33	−14.9	6.2	56.1
10% MNAR 40% MAR	0.39	0.03	94.7	99.3	0.32	−17.2	3.6	57.8
0% MNAR 50% MAR	0.39	−0.05	95.2	99.6	0.36	−8.3	48.4	69.8
***N* = 200**								
50% MNAR 0% MAR	0.39	−0.08	94.4	100.0	0.39	−0.4	94.5	95.0
40% MNAR 10% MAR	0.39	−0.04	95.3	99.9	0.38	−1.13	93.4	79.1
30% MNAR 20% MAR	0.39	−0.0004	95.3	99.9	0.35	−11.1	33.9	65.7
20% MNAR 30% MAR	0.39	0.04	94.9	100.0	0.32	−17.6	4.5	66.8
10% MNAR 40% MAR	0.39	0.08	94.8	100.0	0.33	−15.6	9.3	66.4
0% MNAR 50% MAR	0.39	0.02	95.1	99.9	0.36	−6.8	54.1	78.8
***N* = 100**								
50% MNAR 0% MAR	0.39	−0.1	93.8	100.0	0.39	−0.6	93.7	95.6
40% MNAR 10% MAR	0.39	−0.04	94.2	100.0	0.39	−1.3	92.2	87.5
30% MNAR 20% MAR	0.39	−0.05	93.9	100.0	0.35	−9.9	55.4	82.7
20% MNAR 30% MAR	0.39	−0.01	93.9	100.0	0.33	−15.2	25.2	80.0
10% MNAR 40% MAR	0.39	0.03	93.6	100.0	0.34	−12.1	40.1	81.2
0% MNAR 50% MAR	0.39	0.06	94.9	100.0	0.37	−4.7	79.8	85.2

*NOTE: N*: sample size (number of mice) of the simulated datasets. Mean: average slope estimate of the 1,000 datasets. Bias (%): (slope_estimate_ − slope_true_)/slope_true_*100. Coverage (%): proportion of times the 95% confidence interval of the slope estimated by the model contains the true value. Convergence (%): number of times the statistical model has converged.

## Discussion

In MCTs, antitumor efficacy is evaluated by modeling tumor growth using statistical models for longitudinal data. These models must handle the early dropout induced by the death of mice to avoid survivor bias in analyses over time. Using an example of a MCT with a high rate of dropout due to death during the follow-up, as well as a simulation study, we compared the results of two statistical modeling methods, mixed models and joint models, described in the literature for the analysis of this type of data. We showed, with the analysis of the real dataset and the associated complementary analysis, that the joint model can lead to biased estimates of the antitumor efficacy of a treatment in the presence of a high proportion of dropouts that were explained by the observations, that is MAR. Moreover, the additional simulation study confirmed that the bias is due to the presence of the MAR missing dropout mechanism and showed that this bias is all the higher in the presence of competing dropout mechanisms MAR and MNAR. This problem has previously been identified, in an Human Immunodeficiency Virus (HIV) context, in simulation studies in the presence of different study dropout mechanisms (MAR, MNAR and competing dropout mechanisms MAR and MNAR with a predominance of MAR dropouts; refs. 10, 17).

Joint models including observations to predict death in addition to the current level of biomarker (in our use case tumor volume) have been developed to tackle this problem of biased estimation for MAR situations (10, 17). However, joint models are complex in terms of the number of parameters to be estimated, and it is therefore only possible to use them in the context of MCTs when there is a relatively large amount of data. Indeed, our simulation study showed higher proportions of nonconvergence of the joint models than the mixed models. The nonconvergence of the joint model observed in the simulations corresponds to the worst case. Indeed, for a real dataset, this convergence could probably be improved by specifying some parameters as example initial and/or control values of the convergence algorithm. In addition, joint models can be parametrized in various ways which can lead to misspecification of the model and biased estimates.

In the context of MCT analysis, it is therefore important to record the reasons for dropouts and especially the causes of death, to inform the choice of the statistical model to be used for the analysis of this type of data in particular with many dropouts. In the context of many dropouts due to mouse sacrifices related to too large and therefore unethical tumor size, we recommend using linear mixed models. Furthermore, in the case where in each treatment arm there are several mice from the same PDX [MCT setting as opposed to the single mouse trial (1 mouse per PDX in each treatment arm)], we recommend the use of nested random effects linear mixed models.

For the additional analysis, we created a database containing only MNAR study dropouts by making the tumor growth measurements leading to death unobservable. An alternative would have been to create a database with MNAR study dropouts by randomly drawing MNAR study dropouts from the original dataset. However, in the original database, given the low number of deaths leading to MNAR study dropouts (*N* = 12), this strategy did not seem adequate.

For our analyses, we assumed an exponential tumor growth by applying a logarithmic transformation to the data. We have shown that within our framework of the statistical regression model with change in slopes, there was a good fit of the model to the data under this assumption. However, it should be noted that other more complex forms of tumor growth and/or more complex treatment effects could be estimated using mechanistic models (18). As our present work focuses on the nature of missing data, the choice of the use of a joint model or not would apply also to other models capturing more complex growth dynamics.

In conclusion, the joint models are often recommended in the literature to deal with informative (MNAR) or noninformative (MAR) study dropout. However, these models are valid only under the assumption of a correct specification of the study dropout mechanism. Here we illustrate that this methodology can provide biased estimates under certain MAR study dropout mechanisms and under competitive risk of MAR and MNAR study dropouts. We recommend that a linear mixed model is preferred for MCT analyses.

## Supplementary Material

Supplementary Data S1 Definition of summary measures of tumor growths used MCT endpoints in the literature.

Supplementary Data S2 Details on statistical modeling strategy.

Supplementary Data S3 Linear mixed-effects regression model

Supplementary Data S4 Residuals analysis of joint shared random effect model.
