# Supplementary material for: On the Choice of Longitudinal Models for the Analysis of Antitumor Efficacy in Mouse Clinical Trials of Patient-derived Xenograft Models
Source: Cancer Res Commun. 2023 Jan 26;3(1):140–7. doi: 10.1158/2767-9764.CRC-22-0238 (PMC10035449; doi:10.1158/2767-9764.CRC-22-0238)
Supplement: Supplementary Data S1 — Definition of summary measures of tumor growths used MCT endpoints in the literature. [file crc-22-0238-s01.docx]

**S1. Definition of summary measures of tumor growths used as MCT endpoints in the literature**

- TGI: 1- RTV_tr_/RTV_c_ , with Relative Tumor Volume RTV=TV_t_/TV_0_, t: time, tr : treated and c : control
- AUC ratio: AUC_tr_/AUC_c_
- mRECIST defined by Gao and al(2).: as for the clinical RECIST criterion, 4 categories are defined (complete response : mCR, partial response : mPR, stable disease : mSD and progressive disease : mPD) based on best response and best average response thresholds.
