# Supplementary material for: On the Choice of Longitudinal Models for the Analysis of Antitumor Efficacy in Mouse Clinical Trials of Patient-derived Xenograft Models
Source: Cancer Res Commun. 2023 Jan 26;3(1):140–7. doi: 10.1158/2767-9764.CRC-22-0238 (PMC10035449; doi:10.1158/2767-9764.CRC-22-0238)
Supplement: Supplementary Data S2 — Details on statistical modeling strategy. [file crc-22-0238-s02.docx]

**S2. Details on statistical modeling strategy**

Concerning the modelling strategy, first, the Akaike Information Criteria (AIC) of mixed-effects regression models with different times of change in slope were compared. The model with the smallest AIC was the one with a slope change at 4 weeks after treatment initiation (AIC week 3:2322.003, week 4:1606.093, week 5:1835.794). Then, each random effect was tested, namely intercept, slope before and after week 4. The model with the smallest AIC was the complete model with random intercept, slope before and after week 4. Lastly, the nested random effect was also tested by a likelihood ratio test. This effect was significant with a p.value<0.0001.
