# Supplementary material for: On the Choice of Longitudinal Models for the Analysis of Antitumor Efficacy in Mouse Clinical Trials of Patient-derived Xenograft Models
Source: Cancer Res Commun. 2023 Jan 26;3(1):140–7. doi: 10.1158/2767-9764.CRC-22-0238 (PMC10035449; doi:10.1158/2767-9764.CRC-22-0238)
Supplement: Supplementary Data S3 — Linear mixed-effects regression model [file crc-22-0238-s03.docx]

**S3.** **Linear mixed-effects regression model**

Nested random effects

$$Y_{ij}{\sim\beta}_{0}+\beta_{1}t_{ij}+\beta_{2}\left( t_{ij}>c \right)\left( t_{ij}-c \right)+\beta_{3}X_{i}+\beta_{4}D_{1i}+\beta_{5}D_{2i}+\beta_{6}t_{ij}S_{i}{+\beta}_{7}\left( t_{ij}>c \right)\left( t_{ij}-c \right)S_{i}+\beta_{8}t_{ij}D_{1i}{+\beta}_{9}\left( t_{ij}>c \right)\left( t_{ij}-c \right)D_{1i}+\beta_{10}t_{ij}D_{2i}{+\beta}_{11}\left( t_{ij}>c \right)\left( t_{ij}-c \right)D_{2i}+\beta_{12}t_{ij}D_{1i}S_{i}{+\beta}_{13}\left( t_{ij}>c \right)\left( t_{ij}-c \right)D_{1i}S_{i}+\beta_{14}t_{ij}D_{2i}{S_{i}+\beta}_{15}\left( t_{ij}>c \right)\left( t_{ij}-c \right)D_{2i}S_{i}+\gamma_{0i\vee k}+\gamma_{1i\vee k}t_{ij}+\gamma_{2i\vee k}\left( t_{ij}>c \right)\left( t_{ij}-c \right)+\varepsilon_{ij}$$

with i= 1,…, 225 mice, j= 1, …, n_i_ measured at times t_ij_ and k=1, …, 25 PDX models
