# Supplementary material for: On the Choice of Longitudinal Models for the Analysis of Antitumor Efficacy in Mouse Clinical Trials of Patient-derived Xenograft Models
Source: Cancer Res Commun. 2023 Jan 26;3(1):140–7. doi: 10.1158/2767-9764.CRC-22-0238 (PMC10035449; doi:10.1158/2767-9764.CRC-22-0238)
Supplement: Supplementary Data S4 — Residuals analysis of joint shared random effect model. [file crc-22-0238-s04.docx]

**S4. Residuals analysis of joint shared random effect model**


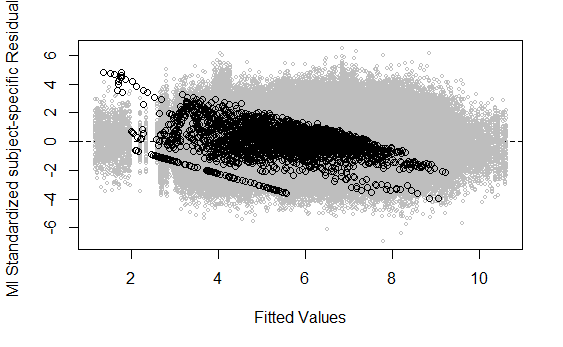


Residuals analysis of the joint shared random effect model with multiple imputation (in grey) to tackle the problem of dropout during the follow-up.
